# Chloroform

**Published:** 1862-05

**Authors:** 


**CHLOROFORM,** to cause insensibility while undergoing painful operations, can never be used with perfect safety. In any given case, the most skillful and experienced administrator can not vouch that the patient will not be dead in ten minutes. No well-attested case has ever come to our notice where fatal results have followed the use of "sulphuric ether" for the same purpose. It has a bad smell, and a large quantity has to be used; but these objections are trifling when the difference is between perfect safety and possible death within a dozen minutes.

